# Skin Injuries Reduce Survival and Modulate Corticosterone, C-Reactive Protein, Complement Component 3, IgM, and Prostaglandin E_**2**_ after Whole-Body Reactor-Produced Mixed Field (n + **γ**-Photons) Irradiation

**DOI:** 10.1155/2013/821541

**Published:** 2013-09-18

**Authors:** Juliann G. Kiang, G. David Ledney

**Affiliations:** ^1^Radiation Combined Injury Program, Armed Forces Radiobiology Research Institute, Bethesda, MD 20889, USA; ^2^Department of Radiation Biology, Uniformed Services University of the Health Sciences, Bethesda, MD 20814, USA; ^3^Department of Medicine, Uniformed Services University of the Health Sciences, Bethesda, MD 20814, USA

## Abstract

Skin injuries such as wounds or burns following whole-body *γ*-irradiation (radiation combined injury (RCI)) increase mortality more than whole-body *γ*-irradiation alone. Wound-induced decreases in survival after irradiation are triggered by sustained activation of inducible nitric oxide synthase pathways, persistent alteration of cytokine homeostasis, and increased susceptibility to systemic bacterial infection. Among these factors, radiation-induced increases in interleukin-6 (IL-6) concentrations in serum were amplified by skin wound trauma. Herein, the IL-6-induced stress proteins including C-reactive protein (CRP), complement 3 (C3), immunoglobulin M (IgM), and prostaglandin E_2_ (PGE_2_) were evaluated after skin injuries given following a mixed radiation environment that might be found after a nuclear incident. In this report, mice received 3 Gy of reactor-produced mixed field (n + *γ*-photons) radiations at 0.38 Gy/min followed by nonlethal skin wounding or burning. Both wounds and burns reduced survival and increased CRP, C3, and PGE_2_ in serum after radiation. Decreased IgM production along with an early rise in corticosterone followed by a subsequent decrease was noted for each RCI situation. These results suggest that RCI-induced alterations of corticosterone, CRP, C3, IgM, and PGE_2_ cause homeostatic imbalance and may contribute to reduced survival. Agents inhibiting these responses may prove to be therapeutic for RCI and improve related survival.

## 1. Introduction

 Radiation injuries combined with another trauma were observed at Hiroshima and Nagasaki, Japan, where approximately 60% of victims received radiation alone with approximately 40% of victims having other injuries concurrent with radiation damage [1, 2]. After the Chernobyl reactor meltdown, 10% of 237 victims exposed to radiation received thermal burns [3]. In *in vivo* experiments with animals including mice [4–7], rats [8, 9], guinea pigs [10], dogs [11], and swine [12, 13], burns and wounds usually increase mortality after otherwise nonlethal irradiation.

Ionizing radiation perturbs hematopoiesis in bone marrow, which, in turn, depresses the innate immune responses against infectious agents, including production of immunoglobulins, and disturbs the inflammatory responses, including C-reactive protein (CRP), the components of complement, and the normal balance among the inflammatory and anti-inflammatory cytokines and chemokines. Endogenously produced interleukin-6 (IL-6) clearly contributes to the survival of mice recovering from radiation injury [14]. However, the relationship between increases in cytokine concentrations, particularly IL-6, CRP, and complement was not clear under the condition of radiation combined injury (CI). 

C-reactive protein (CRP) is a general stress-response protein, which is produced in response to inflammation. Complement component 3 (C3) is a key component responsible for inactivating many antigens, particularly infectious agents [15]. A rise in concentrations of IL-6 in serum, which is produced predominantly by macrophages [16] and adipocytes [17], leads to increases in CRP [18]. CRP is a 224-residue protein with a monomer molecular mass of 25 kDa. It is produced by the liver and binds to phosphocholine on microbes assisting in complement binding to foreign and damaged cells and enhancing phagocytosis by macrophages expressing CRP receptors. It is used mainly as a marker of inflammation. Apart from liver failure, there are few known factors that interfere with CRP production [18]. 

C3 plays a central role in the activation of complement system [15]. Its activation is required for both classical and alternative complement activation pathways. Persons who have a C3 deficiency are susceptible to bacterial infection [15]. 

Immunoglobulin M (IgM) antibodies appear early in the course of an infection and usually reappear, to a lesser extent, after further exposure. Due to its polymeric nature, IgM possesses high avidity and is particularly effective at complement activation. By itself, IgM is an ineffective opsonin; however it contributes greatly to opsonization by activating complement and causing C3b to bind to an antigen [19].

Thus, we postulated that IL-6 would enhance the CRP, C3, and IgM responses to radiation and CI. If this postulation is supported, then the estimation of radiation dose and risk assessment using these biomarkers will be inaccurate under the circumstance of CI. 

It is evident that *γ*-irradiation alters corticosterone concentrations in blood [20]. Corticosterone is an adrenal corticosteroid hormone, which contributes to regulation of immune and stress responses in rodents [21]. The dynamics of corticosterone depended on the scheme of irradiation and the time after irradiation [20]. Therefore, it was of interest to determine blood corticosterone concentrations after high linear energy transfer (LET) whole-body irradiation.

Radiation induces infection [22] and prostaglandin E_2_ (PGE_2_) production [19]. PGE_2_ is a primary product of arachidonic acid metabolism and is synthesized in blood-vessel endothelium in response to infection and inflammation, and it contributes to modulation of inflammation [23]. However, whether skin injuries prior to radiation would magnify PGE_2_ was not clear. Thus, measuring PGE_2_ in irradiated animals with skin injuries could improve understanding of the dynamic changes of PGE_2_ while offering treatments for nuclear-radiation victims.

Care is needed in assessing CI after *γ*-radiation versus mixed field radiation from a nuclear device. The latter is a more realistic radiation scenario after a nuclear incident. Although there have been many studies on *γ*-radiation combined injury, few reports are available on radiation or radiation combined injury from a mixed radiation environment. Our hypothesis was that skin injuries following mixed field radiation exposure would reduce survival and modulate corticosterone, CRP, C3, IgM, and PGE_2_ concentrations in circulation. Therefore, the purpose of this study was to test this hypothesis. We performed studies of combined injured mice in the AFRRI TRIGA Reactor to simulate radiation emitted by a nuclear device and report changes in CRP, C3, and IgM in mice that were given high LET radiation followed by either nonlethal skin wound or burn trauma that would likely occur after a nuclear radiation incident. The benefit of this report is that understanding the changes in the inflammatory responses, which are induced by endogenous IL-6, will provide insight for developing countermeasures to treat radiation combined injury after a nuclear incident. 

Our institute, the Armed Forces Radiobiology Research Institute (AFRRI), is the only facility in the USA having a TRIGA Reactor capable of producing neutron/gamma-photon mixed field irradiations imitating the radiation environment after a nuclear accident. This report presents data obtained from mice exposed to neutron/gamma-photon mixed field radiation followed by skin wound or burn trauma. We are the first to publish in the radiation research field such novel findings for nuclear radiation environments. We report that either skin wound or skin burn amplified the mixed field radiation-induced pathophysiological changes, thereby, subsequently increasing mortality and proving the above-stated hypothesis.

## 2. Materials and Methods

Research was conducted in a facility accredited by the Association for Assessment and Accreditation of Laboratory Animal Care International (AAALACI). All procedures involving animals were reviewed and approved by the AFRRI Institutional Animal Care and Use Committee. Euthanasia was carried out in accordance with the recommendations and guidance of the American Veterinary Medical Association [24, 25].

### 2.1. Animals

B6D2F1/J female mice were purchased from The Jackson Laboratory (Bar Harbor, ME). The mice were held in quarantine for two weeks. Representative samples were examined by microbiology, serology, and histopathology to assure the absence of specific bacteria, particularly *Pseudomonas aeruginosa*, and common murine diseases. Mice were used when they were 12–20 wk old. Male mice were not used in this study because of aggressive behavior, which in these experiments could lead to further injury to wound sites and enhanced infection. All mice were randomly assigned to experimental groups. Mice were maintained on a 12 h light/dark, full-spectrum-light cycle with no twilight.

Prior to experimental manipulation, hair of the dorsal surface of mice was removed under anesthesia (methoxyflurane inhalation) using electric clippers. Mice were placed in well-ventilated acrylic restrainers for irradiation or sham treatments. Within 1 h after irradiation or sham irradiation, mice were anesthetized by methoxyflurane inhalation, and wounding or sham wounding was performed.

### 2.2. Mixed Field Neutron-Gamma Irradiation

Mixed neutron and *γ* fields were produced by the AFRRI TRIGA (Training, Research, Isotopes, General Atomics) Reactor (maximum steady-state power of 1 MW). The average fluence-weighted energy of the unshielded reactor-produced neutrons is 1.49 MeV, which was reduced by appropriate shielding to a mean fluence-weighted energy of 0.98 MeV. An enriched *D*_*n*_/*D*_*T*_ (0.94) field was produced at 4.2 kW, using 20 cm of lead shielding, and the experiments were 100 cm from the reactor core center. The LD_50/30_ and lower and upper confidence limits for mice irradiated in this field (no skin injury) are 3.93 (3.89–3.96) Gy. Centerlines for irradiations were 120 cm above the floor. Mice were irradiated at 0.38 ± 0.02 Gy/min in ventilated aluminum tubes rotating at 1.5 revolutions per min. Dose rates varied less than 2% over the entire field. The *D*_*n*_/*D*_*T*_ ratio was based upon the paired-ion chamber method, where a tissue equivalent chamber and an Mg-Ar gas-flow chamber had different sensitivities to neutrons and to *γ* photons. The nonhydrogenous chamber measured primarily gamma dose rates. The reactor-produced *γ*-photon energies ranged from 10 keV to 10 MeV. The average gamma energy for these spectra ranged from 0.67 to 1.8 MeV on average, similar to that for ion pairs produced by ^60^Co-*γ*-photons [4–7, 22].

### 2.3. Skin Injuries

Skin surface injuries were inflicted on the shaved dorsal surface of mice anesthetized by methoxyflurane inhalation. Methoxyflurane is analgesic up to 48 h after injury. For nonlethal skin wounds, a double layer of dorsal skin and attached panniculus carnosus, comprising 15% of the total body surface area ((TBSA) approximately 24 mm in length and about 15 mm in width) and located between the shoulders and approximately 20 mm from the occipital bone, was removed before or after irradiation by sliding the loose skin from the body and striking the area with a sanitized steel punch on a sanitized Teflon-covered board. For nonlethal skin burns, a 15% TBSA burn was inflicted before or after irradiation on a template-covered shaved dorsal surface of mice by a 12 s ignition of 95% ethanol. All mice received 0.5 mL of 0.9% NaCl intraperitoneally (i.p.) immediately after each type of skin injury [5]. Mice receiving only sham treatments were manipulated identically to other groups but received no radiation, wounds, or burns. Mice given only wounds or burns were also treated identically to other groups but received no radiation.

### 2.4. Survival

The gross appearance, general health, and survival of each mouse were followed by visual inspection twice daily for 30 days in parallel with other assessments. Euthanasia was carried out when animals showed severe dyspnea and inability to move when stimulated, according to US Department of Defense regulations and the AFRRI Institutional Animal Care and Use Committee pertaining to care and use of animals in research. 

### 2.5. Blood Collection

In a separate experiment, mice were anesthetized with methoxyflurane at various time points after radiation and/or wounding or burning. Blood was collected through cardiac puncture prior to euthanasia. Serum or plasma was prepared for biochemical assays including corticosterone, CRP, C3, IgM, and PGE_2_.

### 2.6. Measurements of Corticosterone, CRP, C3, IgM, and PGE_**2**_

Corticosterone, IgM (Abcam, Cambridge, MA), CRP, C3 (GenWay, San Diego, CA), and PGE_2_ (MyBioSource, San Diego, CA) were measured with commercial ELISA kits following the manufacturer's protocol.

### 2.7. Statistical Analysis

Data are expressed as the mean ± SD. For each survival experiment, 24 mice per group were tested on an individual basis. Survival analyses were performed using a Kaplan-Meier curve and the log-rank test. For each bioassay experiment, 6 mice per group were tested on an individual basis. Each biochemical assay was performed for each blood sample. One-way ANOVA, two-way ANOVA, studentized-range test, and Bonferroni's inequality were used for comparison of groups, with 5% as a significant level.

## 3. Results

### 3.1. Survival after Radiation Combined Injury Depended on Injury Type and Timing of Injury

**Figure 1:**
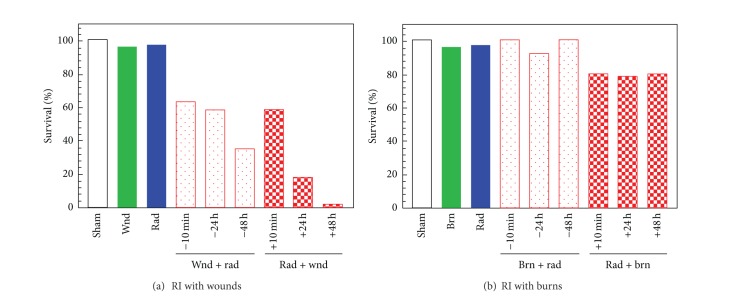
Survival after radiation combined injury depended on injury type and timing of injury. *N* = 24 mice per group and per time point. A representative data set is presented here. No SD is included. Similar results were reproducible in other independent experiments. (a) Skin wounding before or after irradiation reduced 30-day survival after irradiation. Wounding after irradiation decreased 30-day survival more than wounding before irradiation. (b) Skin burning after but not before irradiation reduced 30-day survival after irradiation. wnd: wounding; brn: burning; rad: radiation at 3 Gy (n/n + *γ* = 0.94).

The dose of radiation, which caused 50% mortality in a 30-day period after irradiation (LD_50/30_), was 3.93 Gy for radiation alone, 3.52 Gy for radiation plus burn, and 3.05 Gy for radiation plus wound. When mice received only skin wound, skin burn, or 3 Gy neutron irradiation, mortality was 4%, 4%, and 3%, respectively, during the 30-day period of the study (Figure 1). However, when irradiated mice received skin wounding before or after irradiation, mortality incidence was altered. The magnitude of mortality was affected by the time interval between irradiation and wounding. A longer interval before or after irradiation was associated with greater mortality than were shorter intervals. It should be noted that wounding prior to irradiation attenuated the radiation-induced mortality by 5–41% compared to when wounds were given after irradiation (Figure 1(a)). 

When mice received skin burns after irradiation, mortality increased by 14–20%, but the increase was independent of the time interval between irradiation and burning. When burning was given before irradiation, mortality was only 0–8% (Figure 1(b)). Skin burn was a less severe injury than skin wound. 

Figure 1 was a representative survival study. Similar results were obtained in other independent experiments not included in this study. No standard deviation, therefore, can be inserted in the figure.

**Figure 2:**
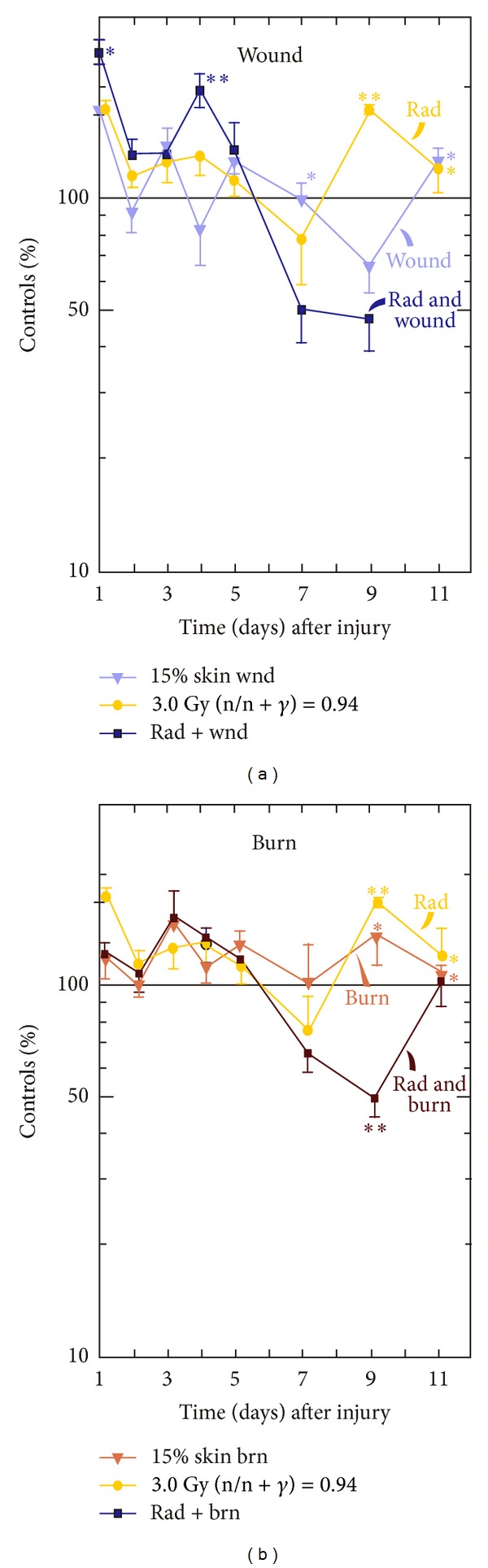
Corticosterone increased then decreased in mouse plasma after irradiation and wounding but not irradiation and burning. *N* = 3–6 per group at each time point. The control corticosterone concentration was 334 ± 24 ng/mL. (a) Skin wound trauma transiently enhanced the radiation-induced increase in plasma corticosterone concentrations at days 1–5 and then reduced it below the baseline at days 5–9. *P < 0.05; **P < 0.01 versus control group. (b) Skin burn trauma reduced the radiation-induced increase in plasma corticosterone concentrations within 1 d, increased at day 3, reduced again between days 7–9, and returned to the baseline line at day 11. *P < 0.05; **P < 0.01 versus control group. wnd: wounding; brn: burning; rad: radiation at 3 Gy (n/n + *γ* = 0.94).

**Figure 3:**
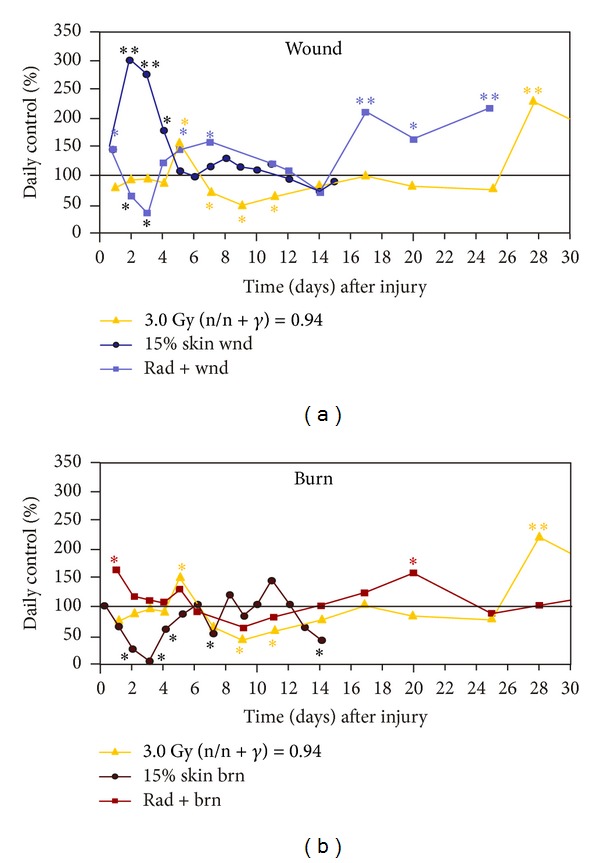
Skin injuries altered CRP responses to radiation. *N* = 3–6 per group and per time point. The control CRP concentration was 4.0 ± 0.1 *μ*g/mL. (a) Skin wound trauma transiently decreased serum CRP concentrations at days 2 and 3, then increased it at days 4–7, reduced to baseline, and rose again at days 16–24. *P < 0.05; **P < 0.01 versus control group. (b) Skin burn trauma increased serum CRP concentrations at days 1, 5, and 20 after irradiation. *P < 0.05; **P < 0.01 versus control group. wnd: wounding; brn: burning; rad: radiation at 3 Gy (n/n + *γ* = 0.94).

**Figure 4:**
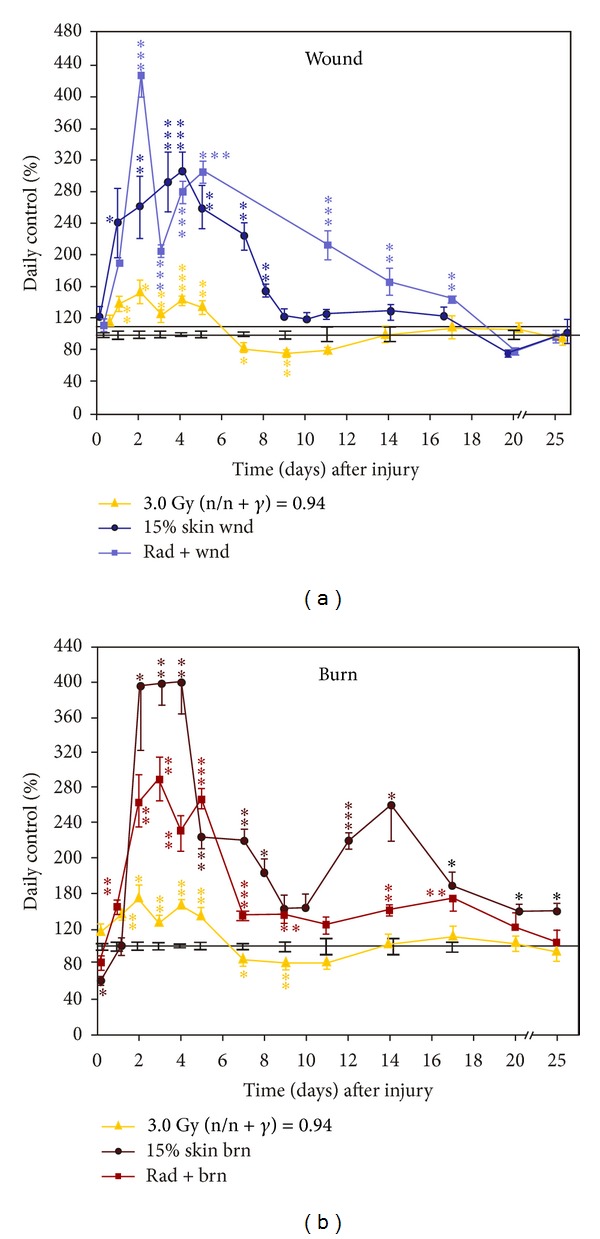
Skin injuries altered C3 responses to radiation.  *N* = 3–5 per group and per time point. (a) Skin wound trauma enhanced and sustained the radiation-induced increase in serum C3 concentrations. The control C3 concentration was 325 ± 10 *μ*g/mL. *P < 0.05; **P < 0.01; ***P < 0.001 versus control group. (b) Skin burn trauma increased and sustained serum C3 concentrations after irradiation. The control C3 concentration was 310 ± 11 *μ*g/mL. *P < 0.05; **P < 0.01, ***P < 0.001 versus control group. wnd: wounding; brn: burning; rad: radiation at 3 Gy (n/n + *γ* = 0.94).

**Figure 5:**
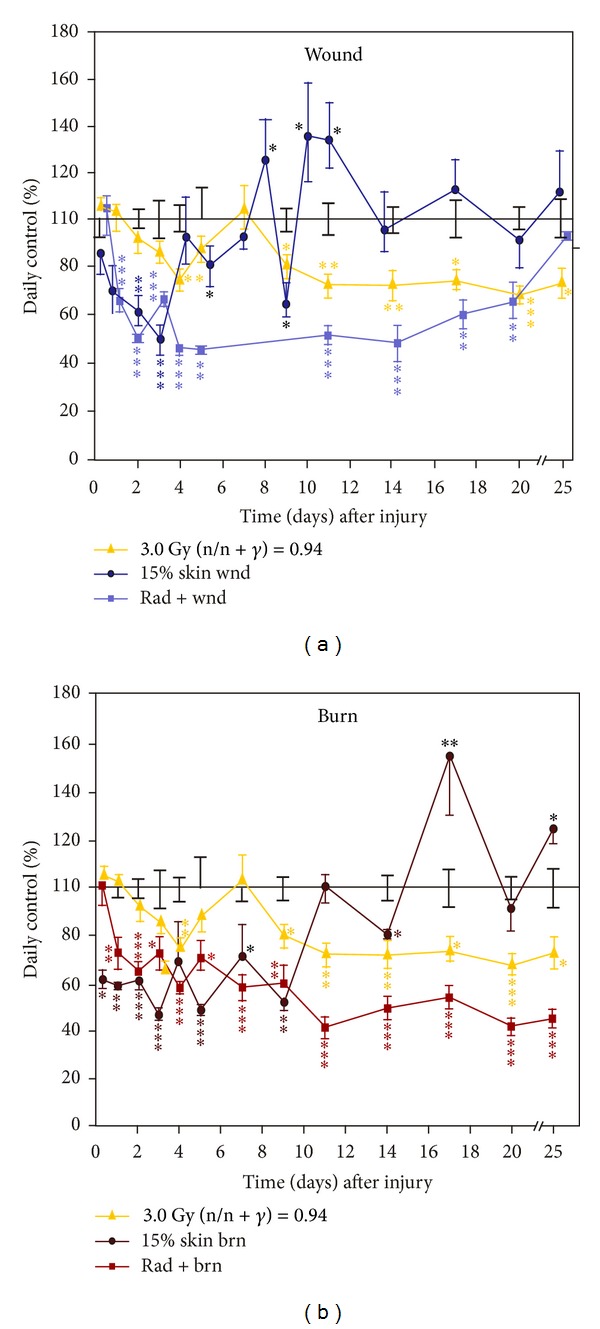
Skin injuries enhanced the radiation-induced decrease in IgM concentrations. *N* = 6 per group and per time point. (a) Skin wound trauma enhanced and sustained the radiation-induced decrease in serum IgM concentrations. *P < 0.05; **P < 0.01; ***P < 0.001 versus control group. (b) Skin burn trauma decreased and sustained serum IgM concentrations after irradiation. *P < 0.05; **P < 0.01, ***P < 0.001 versus control group. wnd: wounding; brn: burning; rad: radiation at 3 Gy (n/n + *γ* = 0.94).

**Figure 6:**
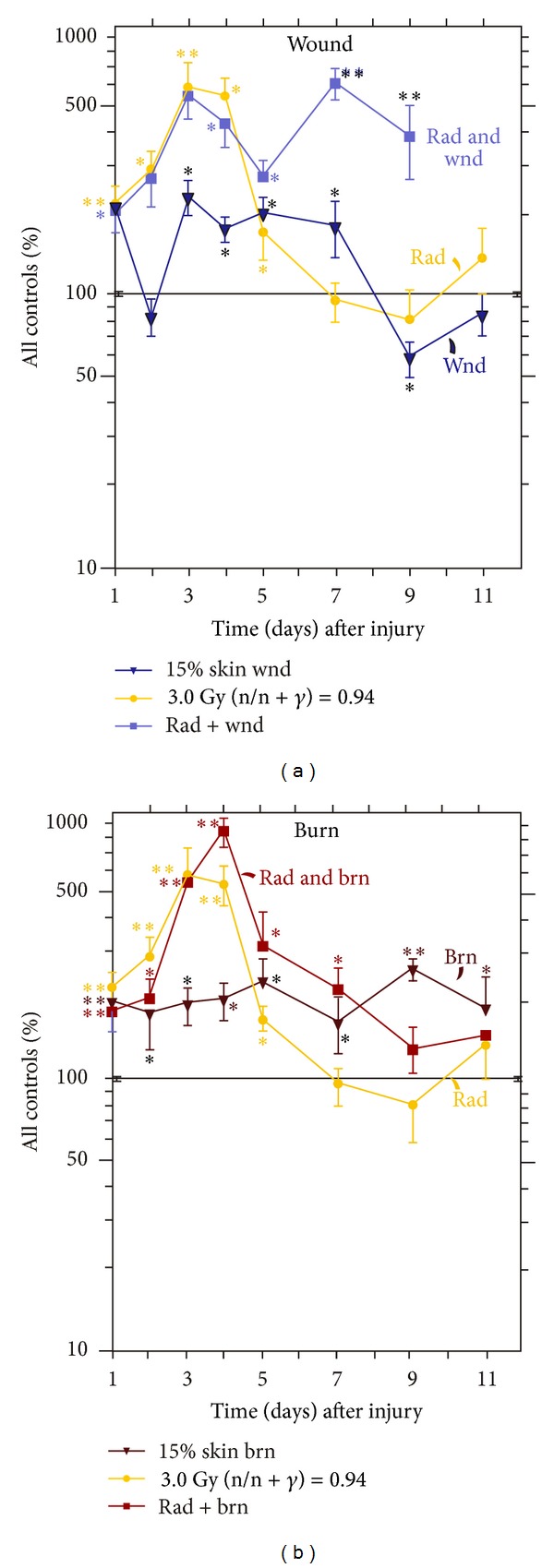
Skin injuries sustained prostaglandin E_2_ response to radiation. *N* = 2–6 per group and per time point. The control PGE_2_ concentration was 15 ± 2 pg/mL. (a) Skin wound trauma sustained the radiation-induced increase in PGE_2_ concentrations. *P < 0.05; **P < 0.01 versus control group. (b) Skin burn trauma transiently enhanced serum PGE_2_ concentrations after irradiation. *P < 0.05; **P < 0.01 versus control group. wnd: wounding; brn: burning; rad: radiation at 3 Gy (n/n + *γ* = 0.94).

The time interval of 10 min between irradiation and subsequent skin injuries was used for the following experiments in order to measure concentrations of corticosterone, CRP, C3, IgM, and PGE_2_ at various time points with sufficient statistical power (Figures 2–6).

### 3.2. Skin Injuries Altered Radiation-Induced Corticosterone Stress Responses

Corticosterone is the main glucocorticoid involved in regulation of stress responses [21]. To determine whether radiation, wound, burn alone, or their combination generated stress, corticosterone concentrations in plasma were measured. Irradiation or wounding induced similar acute increases in corticosterone concentrations within 1 day, whereas irradiation-wound combination increased more corticosterone than either one alone. Corticosterone concentrations returned to baseline in irradiated mice and in wounded mice and remained at baseline during the next 8 days. At day 9, irradiation increased corticosterone concentration again, whereas wounding reduced corticosterone below baseline but rose again at day 11 (Figure 2(a)). In contrast, corticosterone concentrations in irradiated-wounded mice remained above baseline until day 7 and then below baseline (Figure 2(a)).

In contrast to wounds and radiation, burns alone did not acutely increase corticosterone until day 3. At day 9, burning increased the corticosterone concentration similar to irradiation at days 3–5 and 9. A synergistic increase in corticosterone was not observed after the irradiation-burn combination. Instead, at day 7, the corticosterone concentration was below but returned to baseline at day 11 (Figure 2(b)). 

### 3.3. Skin Injuries Altered CRP Responses to Radiation

Because radiation increased CRP in anemic cancer patients [26], CRP in serum was measured in irradiated mice. Irradiation alone transiently increased CRP concentrations at day 5 that returned to baseline quickly and remained low but rose again at day 28. Wounding alone acutely increased CRP concentrations that returned to baseline at day 5. The radiation-wound combination induced a delayed rise at days 4–9 and 17–24 that remained elevated (Figure 3(a)). In contrast, burning alone acutely reduced CRP concentrations that returned to baseline at day 6. The radiation-burn combination increased CRP within 1 day, returned to baseline, and then rose again at days 17–20 (Figure 3(b)).

### 3.4. Skin Injuries Altered C3 Responses to Radiation

C3 plays a central role in the activation of complement system [15]. Its activation is required for both classical and alternative complement activation pathways. Persons who have a C3 deficiency are susceptible to bacterial infection [15]. Moreover, it was evident that CI resulted in an early onset of bacterial infection and occurrence of a severe systemic bacterial infection [26]. Therefore, C3 was measured in these animals.

Irradiation increased C3 concentrations in serum to 140 ± 10% of daily control (*P* < 0.05) within 1 day, but returned to baseline at day 7 and stayed below baseline up to day 25 (Figure 4(a)). Wounding acutely increased C3 concentrations to 240 ± 40% (*P* < 0.01), much greater than irradiation alone, within 1 day and stayed at a plateau for 7 days returning to baseline by day 9. However, irradiation wounding magnified C3 concentrations to 430 ± 30% (*P* < 0.0001) that remained above baseline until day 20. 

In Figure 4(b), burning increased C3 within 1 day, reaching a peak of 400 ± 80% (*P* < 0.01) within 2 days, and remained elevated up to day 25. Unlike wounding, irradiation burning increased C3 concentration to 285 ± 25% (*P* < 0.01), for approximately 5 days, but C3 concentration then decreased to a concentration just above the baseline through day 20. 

### 3.5. Skin Injuries Further Decreased IgM Reduction after Irradiation

IgM antibodies appear early in the course of an infection activating complement and causing C3b to bind to the antigen [27]. In our previous work, Gram-positive and Gram-negative bacterial infections [22] were identified as complications in radiation combined injury situations. Therefore, IgM was measured.  Figure 5 depicts that irradiation reduced IgM within 2 days, reached a nadir within 4 days, returning to baseline by day 8 and falling again at day 10, and remained at this low to the end of the observation. Wounding alone reduced IgM to a nadir within 3 days and returned to baseline at day 7 (Figure 5(a)). The radiation-wound combination reduced IgM concentrations even more than radiation alone but to the similar amount as wounding alone. Unlike wounding alone, the reduction was sustained for 20 days. Burn alone reduced IgM concentrations reaching a nadir within 3 days that returned to baseline by day 10, whereas the radiation-burn combination reduced IgM concentrations that were sustained through the reduction to day 25 (Figure 5(b)). 

### 3.6. Skin Injuries Sustained Prostaglandin E_**2**_ Responses to Radiation

Radiation induces infection [22] and PGE_2_ release [19]. To determine whether skin injuries after radiation would change this inflammatory marker, PGE_2_ concentrations were, therefore, measured using commercial ELISA kits. 

Irradiation increased PGE_2_ in irradiated animals and reached a peak of 550 ± 100% (*P* < 0.01) at day 3 that returned to baseline at day 7 (Figure 6). Wounding also increased PGE_2_ by 200 ± 20% (*P* < 0.01) within 1 day and returned to baseline at day 9. The radiation-wound combination increased PGE_2_, reaching a peak of 580 ± 120% (*P* < 0.01) at day 3 that was sustained up to day 9 (Figure 6(a)). 

Burning increased PGE_2_ to 180 ± 20% within 1 day. The increase was sustained for 11 days. The radiation-burn combination increased PGE_2_ release, reaching a peak of 850 ± 150% (*P* < 0.01) at day 4 and returning to the baseline by day 9.

## 4. Discussion

The condition identified as combined injury (CI) was described more than 9 decades ago. Due to continuing concerns for the potential use of a nuclear weapon by terrorists or a rogue nation, attention has been focused on establishing useful animal systems for evaluating the consequences of exposure to radiation in conjunction with injuries associated with nuclear weapon detonation (RCI). Casualties are expected to overwhelm health care facilities, and thus it is imperative to determine (1) the physiologic changes resulting from radiation, tissue injury, and their combinations that lead to morbidity and mortality and (2) countermeasures useful for mass-casualty applications. In the study reported here, we used an enriched field of neutrons to simulate the nuclear weapon detonation combined with skin injuries to monitor the physiological responses and survival of experimental animals. The results were consistent with those in animals, which received a higher dose of pure ^60^Co-*γ*-photon radiation [4–7, 28–31]. 

In our earlier radiation studies, mice received various doses and qualities of X-rays, ^60^Co-*γ*-photons, and reactor-produced mixed field (n + *γ*-photons) radiations given at 0.4 Gy/min. Compared to X-ray irradiation and ^60^Co-*γ*-photon irradiation, as the *D*_*n*_/*D*_*t*_ increased, the relative biological effect (RBE) of neutrons increased and the LD_50/30_ decreased. The addition of a standard sized wound or burn in irradiated mice further increased the RBE and further decreased the LD_50/30_. In all cases, skin wounds subsequent to irradiation resulted in greater 30-day mortality and protracted wound healing compared to skin burns [7]. In contrast to skin injuries after irradiation, skin injuries prior to irradiation decreased mortality. We now postulate that nonlethal skin injuries precondition the body by significantly increasing the basal levels of IL-6, IL-8, IL-10, G-CSF, and GM-CSF [29] so that the self-defensive mechanism is activated to protect against radiation injury. IL-6 induces production of neutrophils [32] and is now known to inhibit TNF-*α* and IL-1 and activate IL-10 and transcription 3 [33, 34].

 Kiang et al. [29, 30] reported that wound trauma magnified *γ*-radiation induced increases in IL-6 concentration in blood and was further enhanced by a subsequent systemic bacterial infection. An increased IL-6 concentration upregulated nuclear factor-interleukin-6 (NF-IL6) expression that is a transcription factor binding the promoter region of the iNOS gene. On the other hand, wound trauma-enhanced systemic bacterial infections [22, 29, 35] activated toll-like receptor-4 ((TLR-4) a receptor of endotoxin) that increased nuclear factor-keppa B ((NF-*κ*B) a transcription factor to both iNOS and IL-6 genes) expression to transcribe the IL-6 gene and the iNOS gene, forming a positive feedback among iNOS, IL-6, NF-*κ*B, and NF-IL6 [29]. 

The magnitude of mortality increased by radiation-wound combination was greater than by radiation-burn combination. It was also sensitive to the time interval between wounding and irradiation, which was not observed in the case of burns. The discrepancies between wounds and burns are not clear but suggest that different mechanisms are involved. We postulate that, because the concentration of increased IL-6 in serum of burned mice [36] was much lower than that in wounded mice [29], the degree of stress produced by wounds was significantly greater than burns which was indicated by lower corticosterone concentrations and a higher survival rate in irradiated-burned mice (Figures 1 and 2). Further studies of burns are warranted to elucidate this view. 

While wound injury, compared to burn injury, had a greater impact on survival, both wounds and burns given after irradiation resulted in serum increases in CRP, C3, and PGE_2_. Decreased IgM production as well as IgG and IgA production (data not shown) along with an early rise in corticosterone followed by a subsequent decrease was noted for each radiation combined injury situation. 

Although mice did not reveal that CRP concentration correlated with radiation dose or time after exposure [37], our data indicated that a transient increase in serum CRP concentrations at day 5 after irradiation was observed. The difference between our data and that reported by others [37] could be due to the different strains of mice studied in the experiments (BALB/c versus B6D2F1). However the time of the transiently increased CRP concentration is consistent with the onset of bacterial infection in irradiated-wounded mice [29]. In the scenario of radiation combined injury, increased CRP by radiation-wound combination and decreased CRP by radiation-burn combination would over- or under-estimate the radiation dose, respectively. The correction factor involved with CRP as a biomarker for assessing radiation doses cannot be ignored and should be kept in mind. CRP is also recognized as a biomarker of inflammation because it is a result of a rise of IL-6, a proinflammatory mediator [38, 39]. The time of increased CRP concentrations is consistent with the time course of bacterial infection and elevated cytokine concentrations [29, 35]. Therefore, an inhibitor of CRP such as sitagliptin [40] might serve as a countermeasure for either radiation injury or radiation combined injury. On the other hand, CRP promotes binding of complement to microorganisms and enhances phagocytosis by CRP receptor-positive macropahges [38]. Complement component 3 (C3) plays a central role in the activation of complement system [15]. People with C3 deficiency are susceptible to bacterial infections [15, 41]. In addition, increases in PGE_2_ are important for combating radiation combined injury because inhibition of COX-2 by celecoxib and meloxicam exacerbated mortality induced by radiation combined injury [42]. Then, the possibility that these increases in CRP, C3, and PGE_2_ may be self-defense responses and are beneficial and desirable cannot be ruled out. 

Skin injuries decreased IgM concentrations in serum, which is undesirable due to colonization of the injury with bacteria. IgM antibodies rise early in the course of an infection and contribute in activating complement and causing C3b to bind to bacteria [27]. A decreased IgM concentration suggests a reduction of IgM-producing B cells and complement activity, which would impede the host's ability to combat bacterial infections caused by radiation combined injury. This decrease could abrogate the benefit provided by increased CRP, C3, and PGE_2_.

Investigators studying high LET irradiation combined injury also face many challenges. Perhaps the most important are (1) finding effective countermeasures promoting short-term survival, (2) evaluating the consequences of partial body irradiation along with tissue injuries, and (3) developing a skin injury situation for testing in animals. Along this line, it was determined that WR-151327, an aminothiol, increased survival of mice from radiation combined injury in which the LD_50/30_ was increased over that for nontreated mice [43]. However, it is well known that aminothiols as a class have significant toxicity [44] and therefore would have limited practical use.

It is evident that biodosimetry has been focused on low LET irradiation. There are no data available to assess high LET irradiation doses, and no biodosimetry comparisons can be made between the two. Therefore, it is unknown if the biodosimetry data from low LET irradiation will be relevant to assess high LET irradiation doses. The literature on pathophysiological studies including signal transduction pathways after high LET irradiation is very limited as well. Moreover, effects of the high LET irradiation at various radiation dose rates may need to be investigated, even though it is likely that there may be no dose-rate effect unless at very high dose rates [45].

In summary, nonlethal skin injuries prior to irradiation increased survival from high LET irradiation, whereas these skin injuries after irradiation reduced survival from high LET irradiation. The magnitude of survival was sensitive to the time interval between irradiation and skin wounding but not skin burning. Skin wounding but not burning acutely enhanced radiation-induced corticosterone concentrations, but both types of skin injuries then decreased the concentrations below the baseline. Skin wounds increased CRP, but skin burns decreased CRP immediately after irradiation. However, both skin injuries acutely enhanced radiation-induced C3 increases, IgM decreases, and PGE_2_ increases. These alterations were sustained during the observation period. 

The severity of injury manifested immediately after irradiation combined with skin injuries and prognosis for survival depends upon a complex matrix of factors including many physiological endpoints. Changes in the parameters measured in this study support not only early intervention with intensive therapy to strengthen chances of survival but also the concept of successful therapy, which may be adjusted to conserve critical medical resources. Our results suggest that RCI-induced alterations on corticosterone, CRP, C3, IgM, and PGE_2_ cause homeostatic imbalances and may contribute to reduced survival. Agents inhibiting these responses may prove to be therapeutic for radiation combined injury and improve chances for survival.

## Abbreviations

LET: Linear energy transfer
CRP: C-reactive protein
C3: Complement component 3
IL-6: Interleukin-6
IgM: Immunoglobulin M
rad: Radiation
wnd: Wound
brn: Burn
RCI: Radiation combined injury
TBSA: Total body surface area
VSD: Veterinary Science Department
AFRRI: Armed Forces Radiobiology Research Institute.
